# Movements of Individual Digits in Bimanual Prehension Are Coupled into a Grasping Component

**DOI:** 10.1371/journal.pone.0097790

**Published:** 2014-05-28

**Authors:** Frank T. J. M. Zaal, Raoul M. Bongers

**Affiliations:** Center for Human Movement Sciences, University Medical Center Groningen, University of Groningen, Groningen, The Netherlands; Bielefeld University, Germany

## Abstract

The classic understanding of prehension is that of coordinated reaching and grasping. An alternative view is that the grasping in prehension emerges from independently controlled individual digit movements (the double-pointing model). The current study tested this latter model in bimanual prehension: participants had to grasp an object between their two index fingers. Right after the start of the movement, the future end position of one of the digits was perturbed. The perturbations resulted in expected changes in the kinematics of the perturbed digit but also in adjusted kinematics in the unperturbed digit. The latter effects showed up when the end position of the right index finger was perturbed, but not when the end position of the left index finger was perturbed. Because the absence of a coupling between the digits is the core assumption of the double-pointing model, finding any perturbation effects challenges this account of prehension; the double-pointing model predicts that the unperturbed digit would be unaffected by the perturbation. The authors conclude that the movement of the digits in prehension is coupled into a grasping component.

## Introduction

To pick up an object, we bring our hand to the object while at the same time opening the hand a little wider than the size of the object; opening is followed by the final closure of the hand, resulting in enclosing of the object. The classic understanding of prehension is exactly this: prehension is the act of coordinated reaching and grasping, and has been considered as such in the vast majority of prehension studies (for reviews, for instance, see [1], [2]). An alternative view on prehension is that it should be seen as the combination of the individual movements of the contributing digits. In this view, when an object is picked up between thumb and index finger (i.e., with a pincer grip), prehension should be understood from the individual movements of the thumb and index finger. The latter view on grasping was offered by Smeets and Brenner ([3]; see also [4]). The current study builds on a previous one [5], challenging Smeets and Brenner's *double-pointing* model empirically.

The majority of studies of prehension endorse the classic view that prehension should be seen as the coordinated transport of the hand (*i.e*. the reaching component) and opening and closing of the hand (*i.e*. the grasping component). This characterization of prehension dates back to Jeannerod's seminal studies on reaching and grasping, in which he presented the first prehension kinematics data ([6], [7]; see also [2]). Jeannerod proposed the visuomotor-channels hypothesis (see also [8]), in which reaching and grasping were considered to be independent components of prehension, each acting on different object properties: intrinsic object properties (such as object size) would determine the grasping, whereas extrinsic object properties (such as location relative to the actor) would determine the reaching. One of the arguments for hypothesizing independent reaching and grasping components were the different anatomical structures subserving both components of prehension. A second argument was that the reaching component adapts faster to perturbations of object position than does the grasping component to perturbations of object size ([9]–[12]; but see [13]), suggesting a hierarchy of control (cf. [8], [10]). In the vast majority of the prehension literature, grasping has been studied by considering the kinematics of hand apertures (in a precision grip, the distance between thumb and index finger) and reaching has been studied by examining the kinematics of the wrist. Although the anatomical basis for the independence of reaching and grasping as well as the distinction between intrinsic and extrinsic object properties have been questioned (e.g., see [3]), the important point in the context of the present contribution is that an understanding of prehension as the coordination of a grasping and a reaching component has been widely accepted.

Smeets and Brenner ([3]; see also [4], [14]) proposed an alternative view on prehension. In their view, prehension should be considered as the combined but independent movements of contributing digits. When using a pincer grip, the thumb and index finger would move independently to their respective points of contact with the object. The distance between thumb and index finger can be computed by scientists, but the control of grasping is not concerned with this distance per se, Smeets and Brenner proposed. That is to say, prehension should be seen as the parallel unfolding of two pointing-like movements. One way to test this proposal has been to compare the movements that the digits make during prehension with curved pointing movements to the same end locations [15]–[17]. When making contact with an object in grasping, digits arrive on a path close to perpendicular to the object surface. As a matter of fact, the approach parameter in Smeets and Brenner's double-pointing model quantifies this perpendicularity. Therefore, the comparisons between grasping and pointing digit trajectories has included tasks in which participants had to push or lightly tap a target object at the same position and with the same digit as when they grasped it [17] and the task of grasping an object bimanually with the hands clasped together [16]. When looking at the maximum lateral distances of the digits (the distance from the digit to the line connecting a starting position and the target) and the times that the maximum was reached, Smeets and colleagues concluded that the digit trajectories were similar across tasks, suggesting that prehension movements, in fact, were pointing movements.

While Smeets and Brenner's [3] critique on the visuomotor-channel account was shared by many (e.g., see [18]–[20]), their alternative view was also met with reservations in a number of responses to their original paper. The double-pointing model was deliberately kept simple, but some responses considered it as oversimplifying the act of prehension. For instance, Marteniuk and Bertram [18] argued that the model focused on average trajectories, failing to account for the variability in expression of prehensile patterns (for examples on the different hand aperture profiles within the same task demands, see [21]); Newell and Cesari [19] pointed at the importance of considering the task of prehension within context (for examples on how different follow-up actions systematically affect the kinematics of prehension, see [22]–[24]); also, some [19], [25]–[28] took issue with adopting minimal jerk optimization [29] to model the digit trajectories in the double-pointing account. For instance, the minimal jerk model cannot account for the asymmetrical reaching-speed profiles seen in prehension (e.g., see [30]). In sum, the double-pointing model was met with theoretical concerns. Although we share these concerns, while also agreeing with many of the points of critique on the visuomotor-channels hypothesis as expressed by Smeets and Brenner [3], we felt that the double-pointing model needed an empirical test of its core assumption: the assumption that the digits move independently to their respective target positions. This implies that a perturbation of one of the digit's movement would not have any effect on the other digit's movement (cf. [17], [31]). In the case of using a precision grip, using thumb and index finger, when one of the digits would be forced to change its end position due to a perturbation of the object, the other digit should not be affected. Van de Kamp and Zaal [5] tested this prediction in a setup in which participants were asked to pick up an oblong object, the sides of which could be made to slide in or slide out of a common case. This setup allowed changing the required end position for one of the digits, during the movement, while leaving the conditions for the other digit unchanged. The experiment demonstrated that perturbing one digit, in half of their conditions, resulted in significant adaptations of the other digit, thus violating the assumption of independent movement. This result implies that the double-pointing account of prehension does not hold, van de Kamp and Zaal concluded. Van de Kamp and Zaal's findings are in line with other reports of correlations between thumb and index-finger kinematics in unimanual grasping tasks [16], [32].

Although the correlations between the kinematics of the thumb and index finger, and of the perturbation effects on the unperturbed digit might be taken to indicate that the thumb and index finger do not move independently to their respective end positions (cf. [5], [32]), an alternative explanation is that these digits are controlled independently but that biomechanical or neuromuscular coupling makes that their kinematics are weakly correlated (cf. [14], [16], [17]). If the latter were to be the case, the correlations or perturbation effects should not show up in a task of bimanual prehension (cf. [17]). This account would explain why the correlations were only demonstrated in one of Smeets and Brenner's [16] unimanual tasks and not in their bimanual task. The present study had participants perform bimanual prehension, now allowing each hand to move through space freely without any constraint from the other hand. We applied the same type of perturbations as used by Van de Kamp and Zaal [5]. Finding perturbation effects on the unperturbed digit in our bimanual prehension task would demonstrate a coupling of the digits into a grasping component of prehension, we would argue. This inference would generalize to unimanual grasping under the assumption that bimanual and unimanual prehension share a control regime (cf. [16], [33]).

## Materials and Methods

### Participants

Three men and 17 women (mean age of 29.7 years) volunteered to participate in the study. They were right-handed and had normal or corrected-to-normal vision. The study was approved by the Human Movement Sciences Ethical Board of the University Medical Center Groningen, University of Groningen, and the participants signed for informed consent before entering the study.

### Apparatus

Participants were seated behind a table, on which the target object was placed 35 cm away, along the mid-sagittal plane, from an indicated starting location. The target object was an oblong, black box (4 cm wide, 4 cm deep, and 2 cm high), the sides of which could be made to slide out, independently and very fast (see Figure S1 and Movie S1). Sliding out one side added 1.5 cm to the width of the object. This object was the same as the one used in [5]. The base of the object fitted snugly into a 2-mm deep hole of a 5 mm thick plastic strip of PVC that was affixed to the tabletop (see Figure S1). This prevented the object from moving when one of its sides was made to slide out. Next to the object, on both sides, placed in small holes in the strip of PVC, two pairs of photo cells were positioned under each slider, such that the first photo cell would be covered right after the side started sliding out and the other photo cell would be covered just before sliding out ended (see Figure S1). An Optotrak position sensor was positioned about two meters above the table surface, pointing downward. The Optotrak sampled two IREDs (one on the lower medial corner of the nail of each index finger) at a frequency of 200 Hz. The signals from the photo cells were sampled at 400 Hz by the Optotrak Data Acquisition Unit (ODAU). In a perturbation trial, IRED movement was used to trigger the sliding out of one of the sides of the target object. When the average velocity of the index fingers in the principal reaching direction reached a threshold of 25 mm/s, a trigger was sent to the controller of the target object. It took, on average, 99.7 ms (*SD*  = 2.8 ms) for the left-side slider and 131.8 ms (*SD*  = 3.2 ms) for the right-side slider to start their movement. Sliding out took on average 15.2 ms (*SD*  = 1.0 ms) on the left side and 14.0 ms (*SD*  = 2.0 ms) on the right side of the object.

### Design and procedure

**Figure 1:**
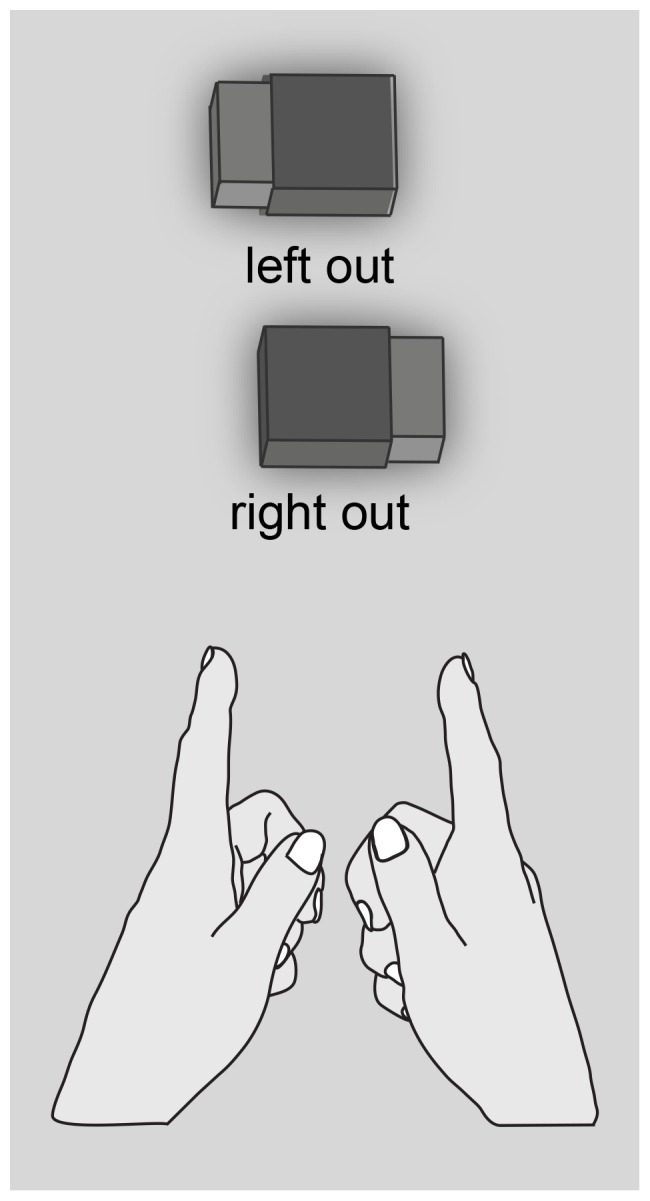
Schematic view of experimental setup and the task. Participants were to grasp the object between the two index fingers. Right after the hand started moving either the left side or the right side of the target object slid out of a common case (see also Movie S1). See text for details.

Participants were asked to perform a prehension movement with the two index fingers (see Figure 1 and Movie S1). They started at the indicated starting location with the pads of the two index fingers touching. After a signal from the experimenter, they were to grasp the target object as fast and as accurately as possible, and hold the object between their index fingers for a second, without lifting it. The design included *static trials*, *perturbation-left trials*, and *perturbation-right trials* (see Figure 1). During perturbation-left and perturbation-right trials, the left side and the right side of the target object, respectively, slid out just after movement initiation. During static trials neither of the sides of the target object did slide out during the trial.

Each participant started with a block of 30 static trials. This block was followed directly by a block of 120 trials, 24 of which were perturbation trials (12 perturbation-left and 12 perturbation-right). The perturbation trials were presented randomly within the block of 120 trials, but were always preceded by at least one static trial.

### Data analysis

Position data was considered in a Cartesian coordinate frame with the origin at the starting location, the y-axis pointing horizontally from the starting location to the middle of the target-object, and the x-axis pointing from the origin to the right hand side. We considered only movement along the horizontal plane. Optotrak position data was filtered with a dual-pass second-order Butterworth filter with a cutoff frequency of 20 Hz. Temporal derivatives of position data were computed with a three-point finite difference algorithm, yielding velocities and accelerations. To define the start and end of each digit's movement, we used a velocity threshold of 25 mm/s. Movement time was the time from the start to the end of the movement.

Our analyses took sets of perturbed and unperturbed trials and compared the digit x-positions at several moments during the movement. The set of unperturbed trials was the set of static trials immediately preceding the perturbation trials under consideration. That is to say, each perturbation-left trial was preceded by a static trial, and the analysis compared the set of perturbation-left trials with the set of these preceding trials. An analogous set of trials preceding the perturbation trials was used in the analyses of the perturbation-right trials. This way, the comparisons were of equal numbers of trials. Statistical analyses were performed on the within-participant averages. We considered digit x-positions at fixed intervals of distance along the y-axis as well as at fixed moments during the movement. More specifically, at each 2 cm along the line of the principal reaching direction we compared the average x-positions of the digits of perturbed and unperturbed trials. Similarly, we compared average x-positions of perturbed and unperturbed trials at each 10% of movement time into the movement. For these comparisons we used paired t-tests, and we report significant differences when *p*-values were below .01.

We did not analyze the data of two participants because one of the IREDs was consistently invisible in the data of one participant and the movements of the other participant were considered too slow (average movement time larger than 1 s); the average movement times (standard deviations) of the remaining participants ranged from 311 (35) to 732 (69) ms and 314 (35) to 721 (57) ms, for the left and right index finger, respectively. As mentioned, we presented 24 perturbation trials to the participants, 12 perturbation-left trails and 12 perturbation-right trials. In the analyses, we did not include the first two perturbation trials of each type. Furthermore, we removed 15 pairs of trials (perturbation trial and preceding trial) because of missing frames during the movement segment, perturbations happening before movement onset, participants not being ready for the trial or starting too early, or responses to the perturbation that resulted in a complete abortion of both digits' movements. In total, we analyzed 345 pairs of trials, 170 of which included perturbation-left trials and 175 of which included perturbation-right trials.

## Results

**Figure 2:**
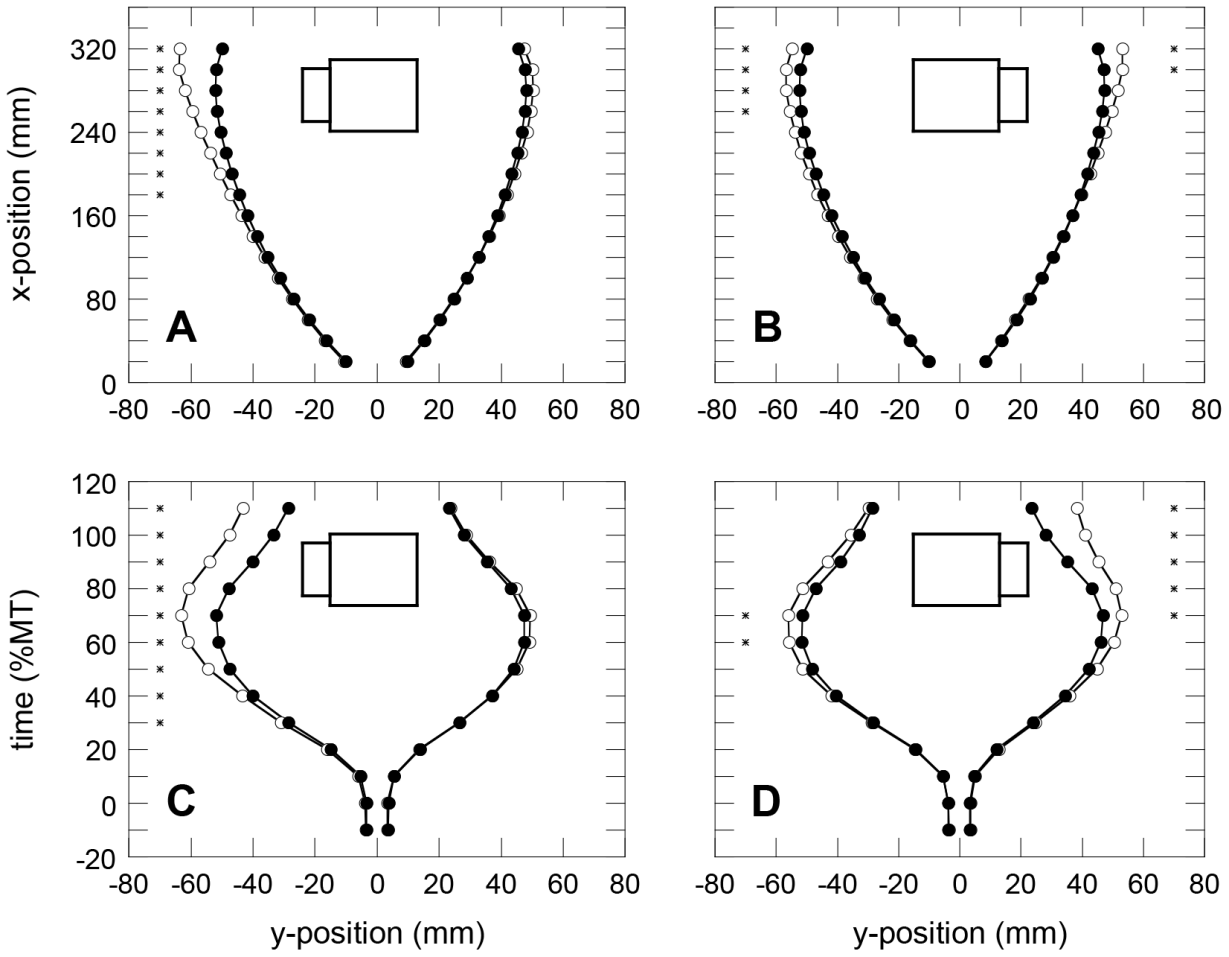
Effects of the perturbation on digit trajectories. Average positions of the two index fingers when unperturbed (solid symbols) and perturbed (open symbols). Panels A and B present the effects of the perturbations as a function of position along the principal reaching direction. Note that the center of the target object was at an x-position of 350 mm. Panels C and D) present the perturbation effects as a function of the moment during reaching. Schematics specify the conditions; Asterisks indicate significant effects of the perturbation (*p*<.01).

Figure 2 shows the average x-positions of the perturbed trials (open circles) as compared to the average x-positions of the unperturbed trials (solid circles) at different moments during the movement. The top panels present the x-positions as function of position along the principal axis of reaching (y-direction). Note that the top panels of Figure 2 do not show average x-positions beyond 32 cm into the reaching, although the object center was at 35 cm. The reason for this was the variability in contact positions along the x-dimension, with a fair amount of trials exhibiting contact at x-position-values lower than 34 cm. This meant that these could not be used to compute average y-positions at 34 cm. To avoid any bias because of this variability in contact points, we did not compute average y-positions at 34 cm. The lower panels of Figure 2 show average y-positions as a function of relative time (percent movement time). To establish that potential perturbation effects were not artifacts, we also considered the averages 10% before movement onset and after the movement ended (determined on the basis of a speed threshold). We used paired t-tests to evaluate any differences of these x-positions. For the perturbation-left condition, these tests demonstrated significant differences only for the left index finger. The first differences appeared at a reaching distance of 180 mm and at 30% into the movement. In contrast, significant differences were present at both digits for the perturbation-right condition. The differences, however, started later in the movement, and slightly earlier for the left index finger than for the right index finger (at a reaching distance of 250 mm and 270 mm, respectively; at 60% and 70% into the movement, respectively).

## Discussion

The objective of the present study was to test Smeets and Brenner's [3] double-pointing model of prehension, now in a task of bimanual grasping. Smeets and Brenner's account is an alternative to the widely-accepted view that prehension is defined as coordinated reaching and grasping. Smeets and Brenner's double-pointing model takes a radically different starting assumption: it assumes that the movements of each of the digits taking part in a prehensile act are controlled independently of the other digits' movements. We tested their hypothesis of independent digit movement by perturbing the future end position of one of the digits in a task in which participants grasped a target object with a pincer grip using the index fingers of their two hands. Van de Kamp and Zaal [5] had used the same target object and the same type of perturbation in unimanual grasping, and had shown effects of perturbing the future end position of one digit on the kinematics of the other digit. That is to say, they found effects of perturbing the end position of the thumb on the kinematics of the index finger, and vice versa. These effects challenged the double-pointing model, Van de Kamp and Zaal claimed. Smeets and Brenner's reply was twofold: first, the analyses presented by Van de Kamp and Zaal did not clearly show what it was in the kinematics that was affected by the perturbation of the opposing digit [14]; second, the effects were small, and could be caused by the biomechanical or neuromuscular coupling between the digits (thumb and index finger of the same hand) while these digits were still controlled independently [14], [17]. Replicating the effects reported by Van de Kamp and Zaal [5] in a bimanual task, in which the object would be grasped with the two index fingers rather than with the thumb and index finger of the same hand, would take away this second concern (cf. [17]). In addition, to counter the first argument, we made an effort to show the effects directly in the paths of the digits (index fingers).

The present study replicated the effects of the perturbation on the unperturbed digit in the bimanual task. When we inspected the lateral positions of the index fingers at fixed intervals along the principal reaching dimension (the y-axis in Figure 2) and at fixed percentages of movement time, we found effects of the perturbation on the lateral positions of the index finger on the unperturbed side (Figure 2). Whereas the argument could be made that the coupling between the digits could be biomechanical in unimanual prehension (cf. [14], [17]), it seems hard to maintain that this is also the case in our task of bimanual prehension, in which the biomechanical coupling between the digits can be safely assumed to be negligible. This implies that the coupling seems to be defined at the level of control. The effects of the perturbations administered in the present study resemble those in a similar study in which participants were asked to point with both index fingers to a set of visual targets on a tabletop [34]. Similar to the present findings, when the position of one of the targets was changed after a participant had started the pointing movement, not only changes in the kinematics on the perturbed side were observed but also on the unperturbed side. However, in contrast to the present results, in that study, the index finger on the unperturbed side moved in the same direction as the index finger on the perturbed side did. For instance, when the target for the right index finger was moved to the right, both index-fingers' trajectories deflected to the right. In the present prehension task, sliding out one side of the target led to outward adaptations of both index-finger trajectories. That is, adaptations in the digits' trajectories were in opposite directions. Perhaps, this difference in the direction of the perturbation effect is related to the fact that objects to be grasped occupy space that cannot be traversed by the digits (i.e. objects can be considered to have obstacle qualities; e.g., see [35]) and because object surfaces are to be approached roughly perpendicularly.

The present findings imply a coupling between the two digits involved in the prehension, such that a perturbation of sliding out one side of the target object leads to outward movement of the digit on the perturbed side as well as on the unperturbed side. A more recent version of the double-pointing model [4], one that uses the concepts of springs and attractors/repellers, includes a spring coupling between the digits. This spring coupling seems to be included to model a biomechanical coupling between thumb and index finger in unimanual grasping rather than a coupling at the level of control. Given the current findings that a coupling between the digits is also present in bimanual prehension, the function of this spring coupling might need to be reconsidered. In fact, the direction of the effects of the perturbation in the current study seems to be hard to reconcile with a simple spring coupling. The perturbation in the current study was designed to move the path of the perturbed digit outwards, orthogonally away from the principal reaching axis; the perturbation was administered by sliding out one side of the target object. The effect that we observed was a movement, also outwards, of the opposing digit. If anything, a spring coupling would result in inward rather than outward deflections of the path of the opposing digit. Thus, the effects of the current study indicate a coupling between the digits at the level of control, a coupling that most probably is different from a simple spring coupling.

An interesting aspect of the findings of the current study, as well as of the findings reported in Van de Kamp and Zaal [5], is the asymmetry in the effects of the perturbation. In the current study, the left index finger was the one to respond to perturbations of the opposing digit. The Van de Kamp and Zaal study showed that the perturbation of unimanual grasping (with thumb and index finger of the right hand) also led to asymmetric effects: sliding out the thumb-side of the target resulted in adjustments in both the thumb and index finger kinematics, whereas sliding out the index-finger side of the target only affected the index-finger kinematics. Thus, it seemed that the index finger in unimanual grasping was more susceptible to the perturbations than the thumb. Together these findings suggest an asymmetric role for the digits involved in prehension, and, more specifically, this asymmetry does not seem to be same for unimanual and bimanual prehension.

It might be hypothesized that the asymmetric effects in both unimanual [5] and bimanual prehension are related to the reaching component of prehension. That is to say, prehension could be understood as coordinated reaching and grasping in which the movement of one of the two digits is controlled as reaching end effector (the control of reaching involves bringing this digit to an appropriate contact point) and the other digit's movement is controlled relative to the other one in a grasping component. Whereas almost all prehension studies have looked at the wrist representing the reaching component of prehension, other studies have suggested that the thumb is being controlled in the reaching to grasp with a pincer grip [36]–[39]. The latter proposal would fit half with Smeets and Brenner's [3], [4] double-pointing models. At least one of the digits is controlled in terms of reaching and the other digit is controlled relative to the reaching one as a grasping component. Then, perhaps the thumb represents the reaching of unimanual prehension and the right index finger represents the reaching in bimanual prehension, at least for right-handed persons. This account, however, would not explain the perturbation effects on both index fingers for both perturbations, as found in Van de Kamp and Zaal's [5] task of unimanual prehension. Under the assumption that object-size perturbations do not affect the reaching of prehension, finding perturbation effects on both digits implies that these digits individually do not represent prehension's reaching component.

We have discussed the present findings in terms of coupled digit movements making up the grasping component of prehension. Earlier studies have demonstrated similar couplings in grasping and speech. When participants are holding an object between thumb and index finger, mechanically perturbing one digit results in immediate compensation at the other digit [40]. Also, considering speech production, when participants are asked to produce a specific utterance, mechanically perturbing the lower lip or the yaw leads to instantaneous compensatory movement of the upper lip [41] or both lips [42], respectively, such that speech is preserved. Synergetic couplings such as these might reflect the way that the multiple degrees of freedom available for almost any goal-directed movement are being mastered. Unraveling these synergetic couplings is important for understanding the control and coordination of human movement. When referring back to the task of prehension, the picture becomes the more complicated the more digits are involved in grasping. Considering the sets of coupled digits into virtual fingers [43] that have to contact the object-to-be grasped at opposing sides (the concept of opposition space; e.g., see [32], [44], [45]) seems a promising route to understand the synergetic organization of prehension.

In conclusion, the current study used a perturbation paradigm to test Smeets and Brenner's [3] double-pointing model of prehension. We demonstrated that the digits do not move independently, not in unimanual prehension (cf. [5]) and also not in bimanual prehension. This suggests that grasping is being controlled in prehension rather than individual digit movement. Some individual digits might be important in the control, but future work is needed to provide a comprehensive account of the roles of all digits in two-digit and multi-digit prehension.

## Supporting Information

Figure S1 **The target object.** (A) Overview: The target object positioned in the PVC strip. The red tubing for pressurized air is connected with the casing on the backside of the object. The participant would be facing the front side. The right-side slider (from the perspective of the participant) has moved out of the common case. (B) Front view: This is how participants would see the target object. Note how the object fits in the PVC strip; the two small holes to the left side of the object contain photo cells used for detection of the sliding movement. The slider on the right side covers the photo cells on this side. (C) Top view: Note the positions of the photo cells in the PVC strip. During an experimental trial, the target object would be positioned in the 2 mm deep hole in the PVC strip. (D) Side view: oParticipants would be approaching the target object from the left. The tubing for the pressurized air is located at the backside of the target object.
(PDF)

Movie S1 **Illustration of the experimental setup.** This movie illustrates how one side of the target object slides out right after the participant has started to move. The participant's task is to grasp the target object between the index fingers of both hands.
(MP4)
