# Supplementary material for: Movements of Individual Digits in Bimanual Prehension Are Coupled into a Grasping Component
Source: PLoS One. 2014 May 28;9(5):e97790. doi: 10.1371/journal.pone.0097790 (PMC4037218; doi:10.1371/journal.pone.0097790)
Supplement: Figure S1 — The target object. (A) Overview: The target object positioned in the PVC strip. The red tubing for pressurized air is connected with the casing on the backside of the object. The participant would be facing the front side. The right-side slider (from the perspective of the participant) has moved out of the common case. (B) Front view: This is how participants would see the target object. Note how the object fits in the PVC strip; the two small holes to the left side of the object contain photo cells used for detection of the sliding movement. The slider on the right side covers the photo cells on this side. (C) Top view: Note the positions of the photo cells in the PVC strip. During an experimental trial, the target object would be positioned in the 2 mm deep hole in the PVC strip. (D) Side view: oParticipants would be approaching the target object from the left. The tubing for the pressurized air is located at the backside of the target object. (PDF) [file pone.0097790.s001.pdf]

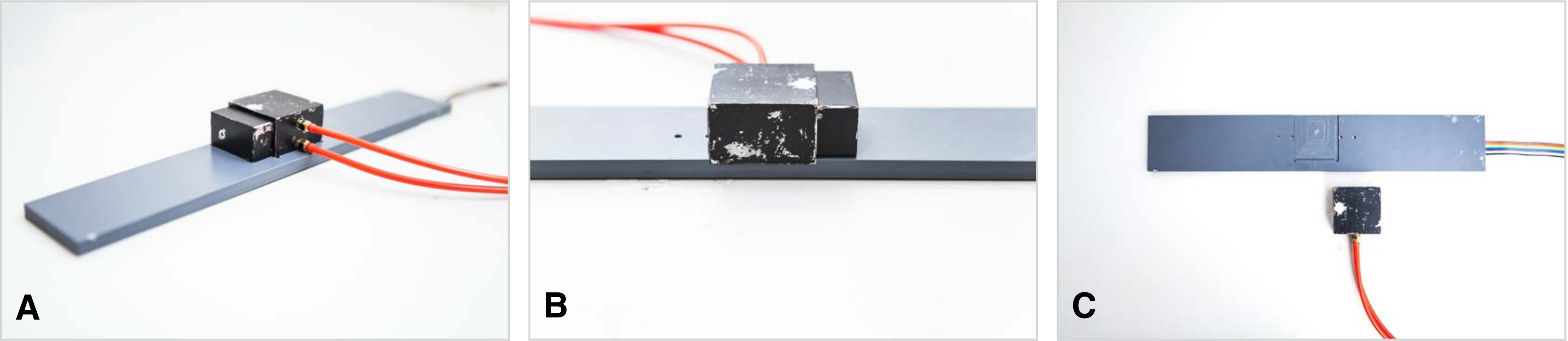

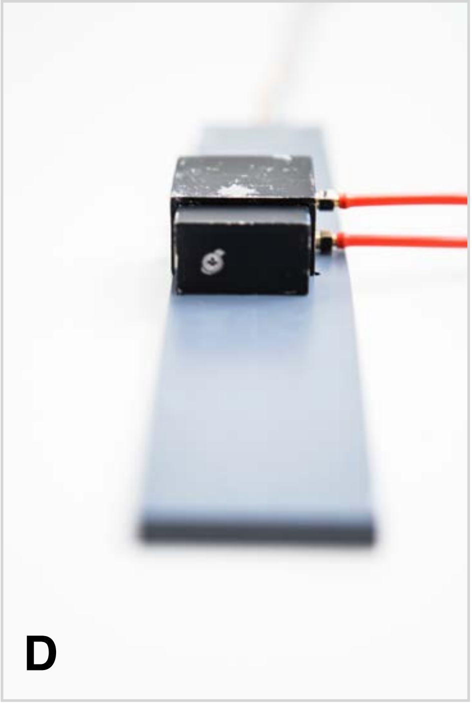

Figure S1. The target object.

(A) Overview: The target object positioned in the PVC strip. The red tubing for pressurized air is connected with the casing on the backside of the object. The participant would be facing the front side. The right-side slider (from the perspective of the participant) has moved out of the common case. (B) Front view: This is how participants would see the target object. Note how the object fits in the PVC strip; the two small holes to the left side of the object contain photo cells used for detection of the sliding movement. The slider on the right side covers the photo cells on this side. (C) Top view: Note the positions of the photo cells in the PVC strip. During an experimental trial, the target object would be positioned in the 2 mm deep hole in the PVC strip. (D) Side view: oParticipants would be approaching the target object from the left. The tubing for the pressurized air is located at the backside of the target object.
